# Bounds on Time Reversal Violation From Polarized Neutron Capture With Unpolarized Targets

**DOI:** 10.6028/jres.110.075

**Published:** 2005-08-01

**Authors:** E. D. Davis, C. R. Gould, G. E. Mitchell, E. I. Sharapov

**Affiliations:** Triangle Universities Nuclear Laboratory, Durham, NC 27708-0308, USA; Triangle Universities Nuclear Laboratory, Durham, NC 27708-0308, USA; Physics Department, North Carolina State University, Raleigh, NC 27695-8202, USA; Triangle Universities Nuclear Laboratory, Durham, NC 27708-0308, USA

**Keywords:** neutrons, resonances, time-reversal

## Abstract

We have analyzed constraints on parity-odd time-reversal noninvariant interactions derived from measurements of the energy dependence of parity-violating polarized neutron capture on *unpolarized* targets. As previous authors found, a perturbation in energy dependence due to a parity (*P*)-odd time (*T*)-odd interaction is present. However, the perturbation competes with *T*-even terms which can obscure the *T*-odd signature. We estimate the magnitudes of these competing terms and suggest strategies for a practicable experiment.

## 1. Introduction

The enhancement factor of a million observed in the 1980s in compound nucleus parity violating observables stimulated great interest in searching for time reversal violation. The enhancement is expected to be present for all symmetry breaking observables in compound nuclear systems, arising as it does from the close spacing and long lifetimes of the states. The largest enhancements were seen in transmission experiments with epithermal neutrons at resonances in nuclei *A* > 100. Despite considerable effort, however, no epithermal neutron transmission test of time reversal violation (*P*-even or *P*-odd) has been carried out, primarily due to difficulties in preparing a suitable spin polarized or aligned nuclear target. For general background on the proposed experiments and the difficulties see [1,2].

For *P*-even time reversal violation, tests with higher energy neutrons have been performed in holmium (*A* = 165) using a nuclear spin aligned target [3]. The experimental precision is high. However, there are no compound nuclear enhancement mechanisms at work, and a 1/*A* suppression factor arises since only the last valence nucleon contributes to the *T*-violating effect. Further improvement with heavy targets and MeV-beams of neutrons therefore appears unlikely. Use of a tensor polarized deuteron target avoids the 1/*A* suppression, and a test using a few hundred MeV polarized proton beam is planned for the COoler SYnchrotron storage ring facility (COSY) at the Institut fur Kernphysik (IKP) Juelich, Germany by the Time Reversal Invariance Test at COSY collaboration (TRIC). The experiment is still under development but does have the potential to make an order of magnitude improvement in sensitivity to the underlying *T*-violating meson exchange coupling constants [4].

Given the difficulties associated with the need for a polarized target in an on-resonance neutron transmission *P*-odd test, it is appropriate to investigate whether other experiments could investigate time reversal violation, taking advantage also of the intense fluxes of neutrons expected to be available from the next generation of spallation neutron sources in the US, Japan and Europe. In the early 1980s, Bunakov and Gudkov [5] and Flambaum and Sushkov [6] noted that measurements with *unpolarized* targets of the *energy dependence* near *p*-wave resonances of *parity-violating* correlations in polarized neutron capture could constrain *P*-odd *T*-odd interactions. Although parity-violating asymmetries of the order of a few percent had earlier been observed in polarized neutron capture, the idea was not pursued further. Instead, in a separate development, the energy dependence of forward-backward asymmetries in *unpolarized* neutron capture was used [7] to look for evidence of *parity-conserving* time-reversal noninvariance. The study was restricted to a single resonance, but demonstrated that the method could in principle yield a competitive bound on the strength of the *P*-even *T*-odd interaction among nucleons if extended to an appropriate sample.

In this paper, we expand on the analysis of *P*-odd *T*-violation suggested in [5,6]. The purpose of the work is to establish to what extent *T*-even contributions may mask the perturbation due to the *P*-odd *T*-odd interaction of interest. Despite uncertainties in the precise values of resonance parameters, the theory of how to model neutron resonance reactions is well enough established to allow us to estimate the order of magnitude of these contributions. We follow the Flambaum and Sushkov model for the energy dependence of the relevant asymmetries.

Our results confirm that there is a shift in the zero of the capture correlation asymmetry from the resonance energy *E_p_*, of order (*v_PT_/v_P_*)*Γ*, where *v_PT_* is the root-mean-square (rms) value of compound nucleus matrix elements of the unknown *P*-odd *T*-odd interaction and *v_P_* is the rms value of compound nucleus matrix elements of the *P*-odd weak interaction. Our results also indicate that, in the epithermal regime, electromagnetic and weak interaction effects give rise to two *T*-even displacements of the zero crossing: one of order $\sqrt{E_{p}(\text{in eV})}10^{-3}\Gamma$ and the other of order (*Γ/D*)*Γ*, where *Γ* is the average width of resonances and *D* is the average spacing between them.

A fuller account, also including analysis of the effects of distant resonances, is published elsewhere [8].

## 2. Two Resonance Analysis

The *P*-odd asymmetry of interest to us measures the strength of the dependence of the differential cross section for the $\left(\vec{n},\gamma \right)$ reaction on the pseudo-scalar ***σ* · *n****_γ_*, where ***σ*** is the transverse polarization of the neutron beam and ***n****_γ_* is the unit vector in the direction of observed photon’s momentum. In the notation of the decomposition of the differential cross section for the $\left(\vec{n},\gamma \right)$ reaction in Eq. (17) of [6], we study the energy dependence of the combination *A* ≡ *a*_9_ − *a*_12_/3, which is precisely the coefficient of ***σ* · *n****_γ_* when all terms in Eq. (17) of [6] are considered. For the sake of definiteness, we restrict ourselves (as do Flambaum and Sushkov in section 3 of [6]) to radiative neutron capture reactions involving:

- a target nucleus with a ${\frac{1}{2}}^{+}$ ground state and a final nucleus with a 0^+^ ground state, and;
- gamma-quanta corresponding to transitions from 1^+^ or 1^−^ states of the intermediate compound nucleus to the 0^+^ ground state of the final nucleus. Then, the general expressions of Appendix A in [6] imply that *A* = *A*^(13)^ + *A*^(24)^, where *A*^(13)^ ≡ 2Re [*V*_1_ (*V*_3_)*] and

A(24)≡2Re[V2(3/2)(V4(3/2))*−V2(1/2)(V4(1/2))*]−12Re[V2(1/2)(V4(3/2))*+V2(3/2)(V4(1/2))*],

*V*_1_, $V_{2}^{\left(j\right)}$, *V*_3_ and $V_{4}^{\left(j\right)}$ being abbreviations for the invariant amplitudes *V*_1_(*E*, 1^+^), *V*_2_(*E*, 1^−^, *j*), *V*_3_(*E*, 1) and $V_{4}^{\left(j\right)}$ (*E*, 1, *j*) of Eq. (15) in [6], respectively.

In the two resonance approximation, only the terms corresponding to the *p*-wave resonance at which the measurement is performed and the nearest 1^+^ *s*-wave resonance (of energy *E_s_* and width *Γ_s_*) are retained in the invariant amplitudes. Thus,

$$V_{1}\approx V_{1}(s)\equiv -\frac{\sqrt{3}}{4k}\frac{g_{n}^{s}g_{M1}^{s}}{E-E_{s}+i\Gamma_{s}/2}$$ (2.1)

$$V_{2}^{\left(j\right)}\approx V_{2}^{\left(j\right)}(p)\equiv -\frac{\sqrt{3}}{4k}\frac{g_{n}^{p}(j)g_{E1}^{p}}{E-E_{p}+i\Gamma_{p}/2}$$ (2.2)

$$V_{3}\approx V_{1}(s)W_{sp}\frac{g_{E1}^{p}/g_{M1}^{s}}{E-E_{p}+i\Gamma_{p}/2}$$ (2.3)

$$V_{4}^{\left(j\right)}\approx V_{2}^{\left(j\right)}(p)W_{sp}^{*}\frac{g_{M1}^{s}/g_{E1}^{p}}{E-E_{s}+i\Gamma_{s}/2}.$$ (2.4)

The notation for the partial width amplitudes ( $g_{n}^{s}$, $g_{M1}^{s}$, etc) differs from that used in [6] (namely, *T_s_*, *A_sf_*, etc). More importantly, we take the interaction matrix element *W_sp_* to include both a *P*-odd perturbation *U* and a *P*-odd *T*-odd perturbation *Ũ*, i.e. *W_sp_* = *u_sp_* + *iũ_sp_*, where *u_sp_* and *ũ_sp_* are real.

Concerning the partial width amplitudes, we assume for the moment that they are all real: $g_{n}^{s}[g_{n}^{p}(j)]$ is the amplitude for capture by the *s*-wave [*p*-wave] resonance of a neutron [of angular momentum *j*]; $g_{M1}^{s}[g_{E1}^{p}]$ is the amplitude for the *M*1 [*E*1] electromagnetic deexcitation of the *s*-wave [*p*-wave] resonance to the ground state. In terms of these partial width amplitudes, the neutron partial widths of the *s*- and *p*-wave resonances are $\Gamma_{s}^{n}={\left(g_{n}^{s}\right)}^{2}$ and $\Gamma_{p}^{n}={\left(g_{n}^{p}\left(\frac{1}{2}\right)\right)}^{2}+{\left(g_{n}^{p}\left(\frac{3}{2}\right)\right)}^{2}$ respectively, and the partial gamma width for the *M*1 and *E*1 transitions to the ground state are $\Gamma_{M1}={\left(g_{M1}^{s}\right)}^{2}$ and $\Gamma_{E1}={\left(g_{E1}^{s}\right)}^{2}$, respectively. Below, the normalized partial width amplitudes $x_{j}^{p}\equiv g_{n}^{p}(j)/\sqrt{\Gamma_{p}^{n}}$ are used.

Substitution of Eqs. (2.1)–(2.4) into *A*^(13)^ and *A*^(24)^ yields

$$\begin{array}{l} A=\frac{3}{{\left(4k\right)}^{2}}\frac{g_{E1}^{p}\Gamma_{p}}{{\left(E-E_{p}\right)}^{2}+\Gamma_{p}^{2}/4}\frac{g_{M1}^{s}\Gamma_{s}^{n}}{{\left(E-E_{s}\right)}^{2}+\Gamma_{s}^{2}/4}\\ \;u_{sp}\left[a_{p}\varepsilon +{\tilde{a}}_{p}\frac{{\tilde{u}}_{sp}}{u_{sp}}+b_{p}\right], \end{array}$$ (2.5)

where, in terms of $z_{p}\equiv {\left(x_{3/2}^{p}\right)}^{2}-{\left(x_{1/2}^{p}\right)}^{2}-\frac{1}{\sqrt{2}}x_{1/2}^{p}x_{3/2}^{p}$, the coefficients *a_p_*, ${\tilde{a}}_{p}$, and *b_p_* are

$$\begin{array}{l} a_{p}=1+z_{p}\frac{\Gamma_{p}^{n}}{\Gamma_{s}^{n}},\;{\tilde{a}}_{p}=1-z_{p}\frac{\Gamma_{p}^{n}}{\Gamma_{s}^{n}}\frac{\Gamma_{s}}{\Gamma_{p}}\\ \text{and}\;b_{p}=z_{p}\frac{\Gamma_{p}^{n}}{\Gamma_{s}^{n}}\frac{E_{p}-E_{s}}{\Gamma_{p}/2}. \end{array}$$

Equation (2.5) demonstrates that a *P*-odd *T*-odd interaction does modify, as claimed in [6], the energy dependence of the *P*-odd asymmetry associated with the pseudoscalar ***σ* · *n****_γ_*.

A signature of this change is its effect on the *location of the zero* in the asymmetry (or, equivalently, *A*). According to Eq. (2.5), the zero is offset from the resonance energy *E_p_* by an amount

$$\Delta E_{p}=-\left(\frac{{\tilde{a}}_{p}}{a_{p}}\frac{{\tilde{u}}_{sp}}{u_{sp}}+\frac{b_{p}}{a_{p}}\right)\frac{\Gamma_{p}}{2}.$$ (2.6)

If we suppose that |*V*_1_| and the $\left|V_{2}^{\left(j\right)}\right|$’s are comparable when *E* ≈ *E_p_* (the parity-mixing essential to the asymmetry under consideration will not be substantial unless this is the case), then

$$\frac{\Gamma_{p}^{n}}{\Gamma_{s}^{n}}\sim \frac{\Gamma_{M1}}{\Gamma_{E1}}{\left(\frac{\Gamma_{p}/2}{E_{p}-E_{s}}\right)}^{2}\sim {\left(\frac{\Gamma }{D}\right)}^{2},$$

where *Γ* is the average width of resonances and *D* is the typical spacing between *J* = 1 resonances, and the following order of magnitude estimates apply: *a_p_* − 1 = *O*(*Γ* ^2^/*D*^2^), ${\tilde{a}}_{p}-1=O(\Gamma^{2}/D^{2})$, and *b_p_* = *O*(*Γ/D*). On omitting terms less than of order (*Γ/D*)^2^*Γ_p_* by at least one order of magnitude [9], the expression for the offset simplifies to

$$\Delta E_{p}=-\left(\frac{{\tilde{u}}_{sp}}{u_{sp}}+b_{p}\right)\frac{\Gamma_{p}}{2}.$$ (2.7)

Observe that Eq. (2.7) implies that Δ*E_p_* ≪ *Γ_p_*/2.

We can accommodate hard sphere phase shifts in our analysis by formally replacing $g_{n}^{s}$ and $g_{n}^{p}(j)$ in Eqs. (2.1)–(2.4) by $g_{n}^{s}e^{i\phi s}$ and $g_{n}^{p}(j)e^{i\phi p(j)}$ respectively. We also have to allow for the fact that the radiative partial width amplitudes are, in principle, complex [10]. To this end, we make the substitutions $g_{M1}^{s}\to \left|g_{M1}^{s}\right|e^{i\phi_{M1}^{s}}$ and $g_{E1}^{p}\to \left|g_{E1}^{p}\right|e^{i\phi_{E1}^{p}}$. In the present two resonance approximation, some of these phases cancel for the combinations of invariant amplitudes appearing in *A* so that, in fact, *A* depends only on the phase differences $\delta_{p}\equiv \phi_{p}\left(\frac{1}{2}\right)-\phi_{p}\left(\frac{3}{2}\right)$ and $\delta_{\gamma }\equiv \phi_{M1}^{s}-\phi_{E1}^{p}$. The coefficients *a_p_*, ${\tilde{a}}_{p}$ *p*, and *b_p_* become

$$a_{p}\left(1+z_{p}^{\prime }\frac{\Gamma_{p}^{n}}{\Gamma_{s}^{n}}\right)\left(\cos\delta_{\gamma }+\frac{{\tilde{u}}_{sp}}{u_{sp}}\sin\delta_{\gamma }\right),$$ (2.8)

$${\tilde{a}}_{p}=\cos\delta_{\gamma }-z_{p}^{\prime }\frac{\Gamma_{p}^{n}}{\Gamma_{s}^{n}}\left(\frac{\Gamma_{s}}{\Gamma_{p}}\cos\delta_{\gamma }-\frac{E_{p}-E_{s}}{\Gamma_{p}/2}\sin\delta_{\gamma }\right)$$ (2.9)

$$b_{p}=-\sin\delta_{\gamma }+z_{p}^{\prime }\frac{\Gamma_{p}^{n}}{\Gamma_{s}^{n}}\left(\frac{E_{p}-E_{s}}{\Gamma_{p}/2}\cos\delta_{\gamma }+\frac{\Gamma_{s}}{\Gamma_{p}}\sin\delta_{\gamma }\right),$$ (2.10)

where $z_{p}^{\prime }\equiv {\left(x_{3/2}^{p}\right)}^{2}-{\left(x_{1/2}^{p}\right)}^{2}-\frac{1}{\sqrt{2}}x_{1/2}^{p}x_{3/2}^{p}\cos\delta_{p}$ The phase difference *δ_p_*, confined as it is to the factor $z_{p}^{\prime }$, which, like *z_p_*, is of order unity, cannot alter the order of magnitude estimates for *a_p_*, ${\tilde{a}}_{p}$, and *b_p_* of the previous paragraph. The dependence on *δ*_γ_ is less trivial, but some consideration of Eqs. (2.6) and (2.8)–(2.10) shows that they can only be reconciled with a measurement of the offset which finds that Δ*E_p_* ≪ *Γ_p_* if |sin*δ*_γ_ | ≪ 1.

If |sin*δ*_γ_ | ≲ *Γ/D*(≪1), then the previous order of magnitude estimates for *a_p_*, ${\tilde{a}}_{p}$, and *b_p_* continue to apply. If, instead, 1 ≫ |sin*δ*_γ_ | ≫ *Γ/D*, then *a_p_* − 1 = *O*(sin^2^ *δ*_γ_), ${\tilde{a}}_{p}-1=O(\sin^{2}\delta_{\gamma })$ and *b_p_* = *O*(sin^2^ *δ*_γ_). *In both cases, Eq. (2.7) holds, it being understood that b_p_ is of order the larger of Γ/D and* sin *δ*_γ_. [In the second case, terms of order *Γ_p_* sin^3^ *δ*_γ_ or smaller have been dropped in Eq. (2.7).]

There is a dearth of information on the order of magnitude of phases like $\phi_{M1}^{s}$ and $\phi_{E1}^{p}$ for epithermal neutron capture in medium-to-heavy nuclei. It has been recognized that they are very small at these energies and so they have been ignored (see, for example, p. 302 in [10]). By adapting the results of [11], we estimate that sin *δ*_γ_ is of order *k/κ_f_* or, equivalently, $\sqrt{E_{p}/S_{n}^{f}}$, where $S_{n}^{f}$ is the neutron separation energy for the ground state of the final nucleus. We also use the fact that the neutron energy *E* of interest is approximately equal to *E_p_* (*k* and *κ_f_* are the neutron wavenumbers corresponding to *E* and $S_{n}^{f}$, respectively).

For nuclei formed in capture on non-fissile spin ${\frac{1}{2}}^{+}$ nuclei of mass number *A* > 100, $S_{n}^{f}$ ranges from about 6 MeV to about 9 MeV. Our order of magnitude estimate of sin *δ*_γ_ thus evaluates to

$$\sin\delta_{\gamma }\sim \sqrt{E_{p}(\text{in eV})}10^{-3},$$

which suggests that *the contribution to* Δ*E_p_ due to the phase difference δ_γ_ is dominant* except in the somewhat unfavourable circumstance (such as with the ^113^Cd target used in [7]) that *Γ/D* ∼ 10^−2^. Even then, *E_p_* has to be less than about 100 eV or so. We find similar results when the effects of distant states are included [8].

## 3. Conclusions

To be of interest as a test of *P*-odd time reversal invariance, data on displacements of zeros in (*n*, *γ*) correlations should comprise measurements at several *p*-wave resonances within a given compound nucleus [12]. For spallation sources, non-fissile nuclei of mass number *A* > 100 emerge as appropriate targets [13]. In practice, the *E_p_*-dependent shift is likely to be the larger of the displacements due to *T*-even interactions: *E_p_* would typically be ≳ 100 eV in any reasonably sized data sample, whereas the choice of target nucleus would almost certainly be such that *Γ/D* < 10^−2^. Taking *Γ* ∼ 100 meV (appropriate to non-fissile *A* > 100 nuclei in the epithermal regime), we expect this *E_p_*-dependent shift (when dominant) to be of order 1 meV. Consequently, one ought to detect non-zero displacements in measurements which can determine the location of zeros with a precision of order 0.1 meV.

When this level of precision cannot be attained and only bounds on shifts in zeros are set, the corresponding bound on the strength of a *P*-odd *T*-odd interaction is not encouraging. One measurement close to threshold (*E_p_* ∼ 1 eV) will not suffice [12]. Several null measurements, which put limits on shifts of slightly more than the 1 meV or so estimated above for the *E_p_*-dependent shifts, would constrain the ratio *v_PT_/v_P_* to be less than of order 10^−2^.

How much better can one do if *non*-null measurements of the displacements of zeros are possible? Individual measurements are, of course, not amenable to quantitative analysis because the precise value of the *T*-even interaction shift estimated in this paper (and other shifts due to effects not considered in this work like non-resonant direct neutron capture) cannot be calculated with any certainty. What, conceivably, could be done is to model the *statistics* of shifts reliably. Values of this shift are drawn from a Cauchy distribution with scale parameter *λ* ≡ *v_PT_/v_P_*. With a large enough sample of non-null determinations of displacements (of zeros) and a sound model for their statistics, it should be possible to extract some information on *λ*. For example, if a statistical analysis could fix a bound on the shift due to the *P*-odd *T*-odd interaction at ten percent of the *T*-even interaction shift (∼10^−2^*Γ*), the corresponding bound on *λ* would be of order 10^−3^. This would be a competitive limit, comparable to the kind of bound it has been suggested could be extracted from the 3-fold transmission test requiring a polarized target [14].

## References

[1] Roberson NR, Gould CR, Bowman JD (1987). Test of Time Reversal Invariance. Neutron Physics.

[2] Lamoreaux SK, Golub R (1994). Phys Rev D.

[3] Huffman PR, Roberson NR, Wilburn WS, Gould CR, Haase DG, Keith CD, Raichle BW, Seely ML, Walston JR (1997). Phys Rev C.

[4] Eversheim PD (1998). Nucl Phys.

[5] Bunakov VE, Gudkov VP (1983). LNPI Preprint 881.

[6] Flambaum VV, Sushkov OP (1985). Nucl Phys A.

[7] Barabanov AL, Sharapov EI, Skoy VR, Frankle CM (1993). Phys Rev Lett.

[8] Davis ED, Gould CR, Mitchell GE, Sharapov EI (2004). Phys Rev C.

[b9-j110-4dav] 9We implicitly assume here that |*ũ_sp_/u_sp_*| or, more precisely, the ratio of their rms values is very much less than unity.

[10] Lynn JE (1968). The Theory of Neutron Resonance Reactions.

[11] Lane AM, Lynn JE (1960). Nucl Phys.

[12] Davis ED, Gould CR (1999). Phys Lett B.

[b13-j110-4dav] 13The level density in the compound nucleus formed after neutron capture is high enough that several *p*-wave resonances can be studied with large neutron fluxes, while *Γ/D*is very much less than unity (because the fission channel is closed).

[14] Koehler PE, Gould CR, Haight RC, Valentine TE, Astrophysics (2002). Symmetries, and Applied Physics at Spallation Neutron Sources.

